# Extraction, Purification and Primary Characterization of Polysaccharides from Defatted Peanut (*Arachis hypogaea*) Cakes

**DOI:** 10.3390/molecules21060716

**Published:** 2016-06-01

**Authors:** Hongzhi Liu, Nan Jiang, Li Liu, Xiaojing Sheng, Aimin Shi, Hui Hu, Ying Yang, Qiang Wang

**Affiliations:** 1 Institute of Food Science and Technology, Chinese Academy of Agricultural Sciences/Key Laboratory of Agro-Products Processing, Ministry of Agriculture, Beijing 100193, China; liuhongzhi@caas.cn (H.L.); jiangnan_fx@163.com (N.J.); liulicaas@126.com (L.L.); s_ilence0101@126.com (X.S.); sam_0912@163.com (A.S.); huhui@caas.cn (H.H.); yingyang_umass@126.com (Y.Y.); 2 Beijing Research Center for Agricultural Standards and Testing, Beijing Academy of Agriculture and Forestry Sciences, Risk Assessment Lab for Agro-Products (Beijing)/Ministry of Agriculture, Beijing 100097, China

**Keywords:** defatted peanut cake, polysaccharide, response surface methodology, purification and characterization

## Abstract

The hot-water extraction, purification and characterization of polysaccharides from defatted peanut cake (PPC) were investigated in this study. A Box-Behnken factorial design (BBD) was used to investigate the effects of three independent variables, namely extraction temperature (X_1_), extraction time (X_2_) and ratio of water to raw material (X_3_). The optimum conditions were 85 °C, 3 h and 20:1 (mL/g) respectively. Regression analysis was done to reveal the experimental results which include 34.97% extraction rate while the value verified under these conditions was 34.49%. The crude PPC was sequentially further purified by Sephadex G-100 chromatography, and one purified fraction was obtained. The PPC purified fraction was characterized by FT-IR, HPAEC; SEC-MALLS. The average molecular weight of the PPC purified fraction was 2.383 × 10^5^ Da. The polysaccharide was mainly composed of glucose, galactose, arabinose and xylose. The PPC have the typical absorption of polysaccharide.

## 1. Introduction

In recent years, peanut yields have increased year by year, especially in China. At 2013, its annual production was approximately 3,600,000 tons, or almost 40% of the global production and ranked first in the world [1]. As one kind of oil crop, 55% of peanuts are used for oil expression and as a result, large amounts of defatted peanut meal are produced. Defatted peanut cake is rich in a variety of active ingredients, with polysaccharide being the second biggest component.

Polysaccharides have attracted much attention with respect to their biological functions such as immunoreactivity [2], anti-tumor [3], anti-inflammatory activity [4] and antioxidant activity [5]. Researchers have successfully extracted polysaccharide from various materials, including plants, animals and microorganisms. However, the polysaccharides from defatted peanut cake (PPC) have not been investigated much yet.

Nowadays, lots of techniques such as hot-water method [6], acid-alkali method [7], enzyme-assisted extraction method [8] and solvent extraction method [9] can be used for the extraction of polysaccharide. The hot-water technology is the main conventional method for the extraction of polysaccharides mentioned in recent studies [10]. Nevertheless, there are few reports regarding the process of polysaccharide extraction from defatted peanut cake using the hot-water method.

Response surface methodology (RSM) is a useful statistical and mathematical technique for optimizing processes [11,12,13]. Through RSM, the optimization of process can be conducted with the minimum experimental trials and the maximum accuracy. Box-Behnken design (BBD), one of the types of RSM, only has three levels and needs fewer experimental trials, so it is widely used by many researchers [14,15]. It is more efficient and easier compared with other designs when the number of factors are less than five.

The main objective of this study was to optimize the hot-water technology conditions for the extraction of polysaccharides from defatted peanut cake. RSM was used to analyze the effects of extraction parameters on the yields of polysaccharides from defatted peanut cake and their interactions. Furthermore, the crude PPC was purified by Sephadex-G100 chromatography. In addition, the crude PPC and its purified fraction were characterized by chemical analysis, Fourier Transform-Infrared Spectroscopy (FT-IR), High-Performance Anion-Exchange Chromatography (HPAEC) and Size Exclusion Chromatography-Multiangle Laser Light Scattering (SEC-MALLS).

## 2. Results and Discussion

### 2.1. The Composition of Defatted Peanut Cake and Its Carbohydrate Content

The composition of defatted peanut cake is shown in Table 1. The protein content in defatted peanut cake was 47.28% and the high protein content may influence the extraction of polysaccharides. This is the reason that we have to remove the protein first. Otherwise, it is evident that there is much polysaccharide in defatted peanut cake.

**Figure 1:**
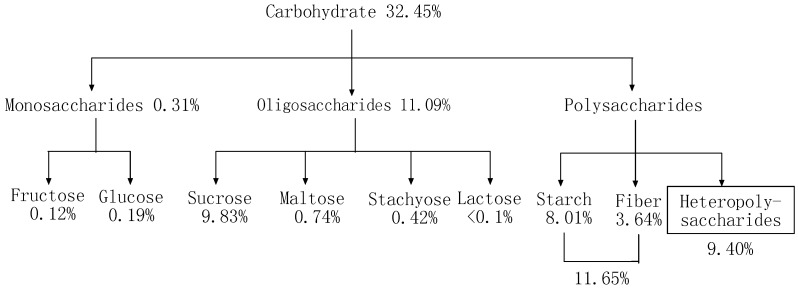
The carbohydrate composition of defatted peanut cake.

Figure 1 shows the carbohydrate composition of defatted peanut cake. Among the sugars, sucrose has the highest content. Previous studies have reported that sucrose, glucose, and fructose can be found in peanut seed, but the proportion of sucrose is the highest [16,17,18,19]. The content of heteropolysaccharides was calculated by subtracting the content of monosaccharides, oligosaccharides, starch and fiber from the total defatted peanut cake sugars. There was a different content of sugars among different peanut cultivars. As well, the oligosaccharide content of peanuts was found to be lower than that reported for soybeans [20,21,22].

### 2.2. Model Fitting and Response Surfaces Analysis

#### 2.2.1. Statistical Analysis and the Model Fitting

The design matrix for the three independent variables including extraction temperature (X_1_), extraction time (X_2_), and ratio of water to raw material (X_3_) together with the response values of each experimental trial are shown in Table 2. Predicted response (Y) for the extraction rate of polysaccharides from defatted peanut cake could be expressed by the following second order polynomial equation in terms of coded values:

Y = 14.11667 + 9.23625X_1_ + 0.695X_2_ − 0.47625X_3_ + 6.570417X_12_ + 0.735X_1_X_2_ − 5.5625X_1_X_3_ − 8.252083X_22_ − 0.36X_2_X_3_ − 0.934583X_32_ (1)

The significance of each coefficient was determined by using the *F*-test and *p*-value as shown in Table 3. The ANOVA of the quadratic regression model demonstrated that the model was highly significant with a very low *p*-value (*p* < 0.0001) through *F*-test. The data in Table 3 also indicate that the variables with the largest effect were the linear terms of extraction temperature (X_1_), extraction time (X_2_), and ratio of water to raw material (X_3_) and the quadratic term of extraction temperature (X_1_ × X_1_), extraction time (X_2_ × X_2_) and ratio of water to raw material(X_3_ × X_3_). Besides, the interaction effects of extraction temperature and ratio of water to raw material (X_1_ × X_3_) were also extremely significant (*p* < 0.0001).

As shown in Table 4, the correlation coefficient (R^2^) was 0.9995, indicating a reasonable fit of the model to the experimental data. Coefficient of variation (CV) represents the degree of precision with which the experiments are compared. A relatively low value of CV (2.68) indicates a better precision and reliability of the experiments carried out. These values indicated that the accuracy and the general availability of the polynomial model were adequate.

#### 2.2.2. Analysis of Response Surfaces

**Figure 2:**
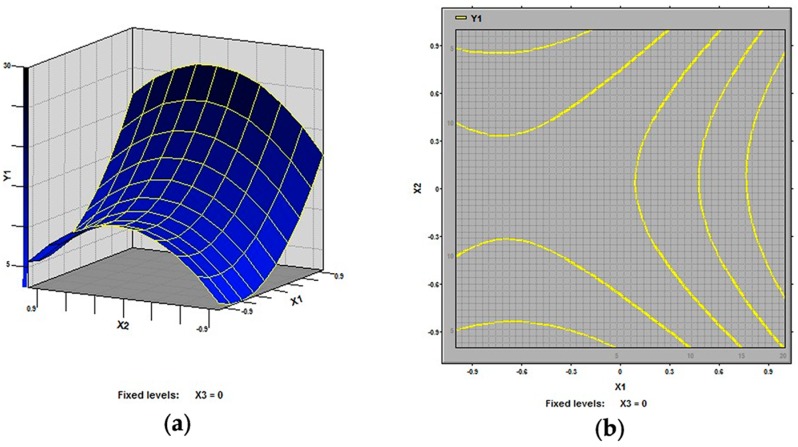
Response surface plot (**a**) and contour plot (**b**) of extraction temperature and extraction time and their mutual interactions

**Figure 3:**
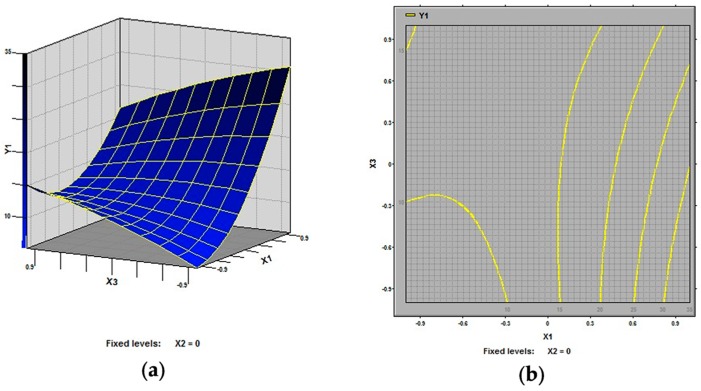
Response surface plot (**a**) and contour plot (**b**) of extraction temperature and ratio of water to raw material and their mutual interactions.

**Figure 4:**
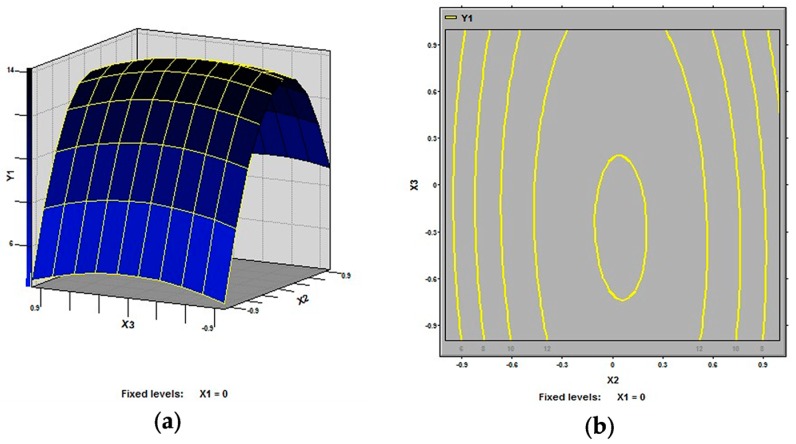
Response surface plot (**a**) and contour plot (**b**) of extraction time and ratio of water to raw material and their mutual interactions.

In this study, our aim was to find the optimized conditions which give the maximum extraction rate of polysaccharides from defatted peanut cake. The 3D response surfaces and 2D contour plots are the graphical representations of regression equation. They provide a method to visualize the relationship between response and each variable and to reflect the interactions between variables. Two variables within the experimental range are depicted in 3D surface plots when the third variable is kept constant at zero level. Different shapes of the contour plots indicate different interactions between the variables, also reflecting whether these interactions between variables are significant or not. The 3D response surfaces and 2D contour plots generated by the model for extraction rate of polysaccharides were displayed in Figure 2, Figure 3 and Figure 4. It is clear that the extraction rate of polysaccharides was sensitive to small changes of the test variables (extraction temperature, extraction time and ratio of water to raw material).

As shown in Figure 2 and Figure 3, all the variables had significant effects on the extraction rate of defatted peanut cake polysaccharides. Specifically, in Figure 2, the extraction temperature (X_1_) demonstrated an exponential increase of the extraction rate in the range of 70–90 °C, while the extraction time (X_2_) also showed an increase in the response in the range of 2–4 h. It is generally believed that the high extraction rate for polysaccharides is mainly due to the high extraction temperature and the long extraction time [23,24,25,26,27]. Figure 3 shows the effect of extraction temperature (X_1_) and ratio of water to raw material (X_3_) on the extraction rate of defatted peanut cake. It was observed that extraction rate increased at first and then decreased with the increase in ratio of water to raw material (X_3_). This could result from that the increase of amounts of solvent molecules also the blend might affect the polysaccharides gelatinization and the rheological properties of the raw material [28,29]. From Figure 4, the extraction rates increased at first and then decreased with the rise of extraction time and ratio of water to raw material while the extraction temperature (X_1_) was fixed at 0 level.

By analyzing the plots, the predicted maximum value (34.97%) of the tested variables for extraction rate, lied in the following condition: extracting temperature (X_1_) 83.7 °C, extracting time (X_2_) 2.8 h, and ratio of water to raw material (X_3_) 18:1 (mL/g). In the optimal conditions, the experimental yield was 34.49% ± 0.3%, which agreed with the predicted value. Therefore, it was confirmed that these conditions were optimal for the extraction rate.

#### 2.2.3. Optimization of Extracting Parameters and Validation of the Model

The suitability of the model equation for predicting the best response values was verified using the selected optimal conditions. The maximum predicted yield and experimental yield of polysaccharides from defatted peanut cake were given in Table 5. Additional experiments for polysaccharides extraction were carried out using the predicted optimum conditions: extraction temperature of 83.7 °C, extraction time of 2.8 h, ratio of water to material of 18:1 (mL/g) and the model predicted a maximum response of 34.97%.

To ensure the predicted result was not biased, experiment rechecking was performed using these modified optimal conditions: extraction temperature of 85°C, extraction time of 3 h, ratio of water to material 20:1 (mL/g). A mean value of 34.49% ± 0.3% (*n* = 3) was obtained, which was in agreement with the predicted value without significant difference (*p* > 0.05) and also demonstrated the validation of the RSM model. The analysis results confirmed that the response model was adequate for reflecting the expected optimization and the model was satisfactory and accurate.

### 2.3. Separation and Purification of the PPC

**Figure 5:**
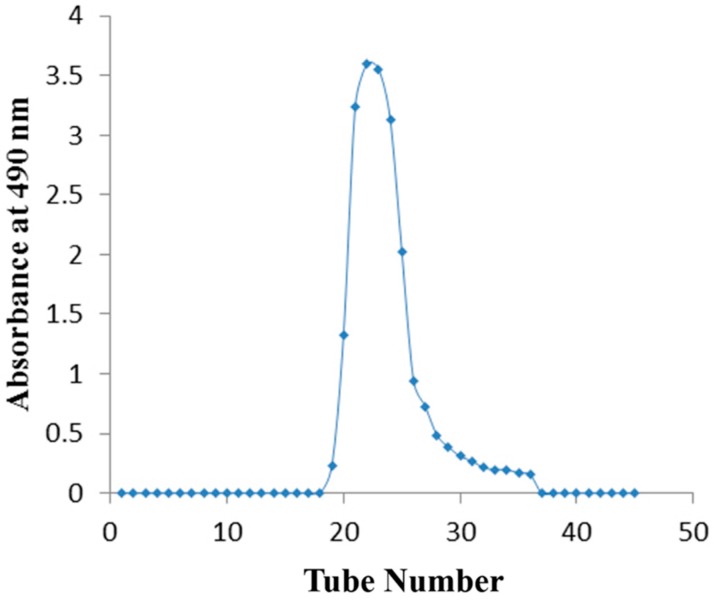
Sephadex G-100 gel filtration chromatography the non-starch PPC.

The water-soluble crude polysaccharide was obtained from defatted peanut cake by hot water extraction, ethanol precipitation, deproteinized by neutral protease, removing the starch and dried under vacuum. The non-starch polysaccharide was purified by preparative Sephadex G-100 gel filtration chromatography to obtain one fraction (Figure 5). The purified polysaccharide fraction showed only one symmetrical peak from gel-filtration chromatography on a Sephadex G-100 column. The results indicated that no other polysaccharide was present in the sample.

### 2.4. Characterization of Polysaccharides

#### 2.4.1. Identification of Polysaccharide Components

Table 6 shows the contents of total sugar, protein, total starch in PPC, non-starch PPC and its purified fraction. The total sugar content in PPC was much higher than non-starch PPC and purified fraction, because of included amounts of starch. In order to remove the starch, α-amylase was used. The total starch in non-starch PPC was 4.19%, and more than 90% of the starch was removed. The total sugar content in the purified fraction was 82.46%, and protein and starch were not detected. These results indicate that the purification process could dramatically reduce the impurities such as starch and proteins in PPC, but the purity of the purified PPC fraction needs to be further improved.

#### 2.4.2. Molecular Weight Determination

For determination of the molecular weight of the PPC purified fraction, the SEC-MALLS method was used. The average molecular weight of the PPC purified fraction was estimated to 2.383 × 10^5^ Da, Mw = 3.418 and the mass fraction was 95%.

#### 2.4.3. Analysis of Monosaccharide Composition

**Figure 6:**
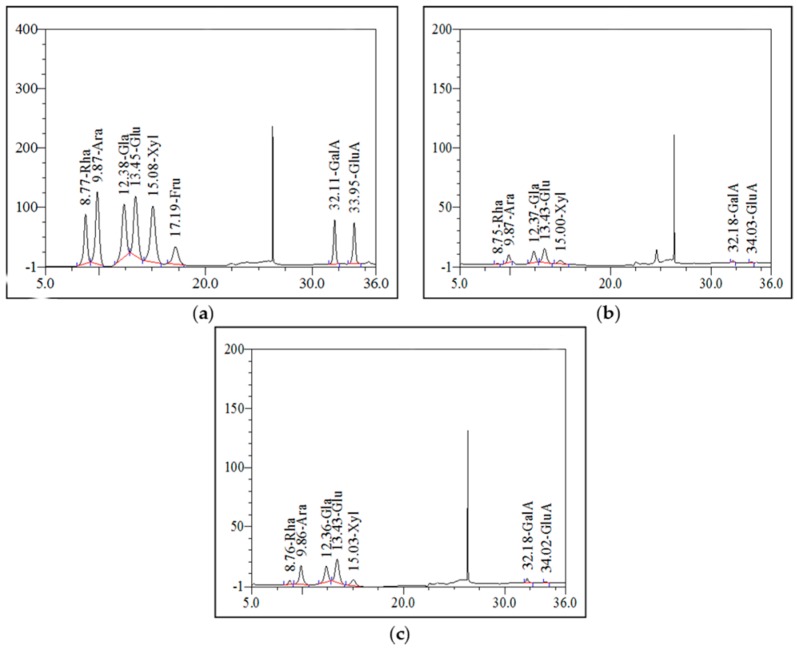
Monosaccharide compositions of the polysaccharides (**a**) standard substance; (**b**) non-starch PPC; (**c**) PPC purified fraction).

The monosaccharide composition of the polysaccharides was analyzed by HPAEC. HPAEC is one of the most suitable methods since it is quantitative and does not require monosaccharide derivatization [30]. The result of HPAEC analysis are depicted in Figure 6. The monosaccharide standards, the non-starch PPC and the PPC purified fraction were mainly composed of glucose, galactose, arabinose and xylose. There were small amounts of rhamnose, galacturonic acid (GalA) and glucuronic acid (GluA). In addition, non-starch PPC was found to be present in a molar ratio of Glu:Gal:Ara:Xyl:Rha:GalA:GluA = 39.30:28.97:14.81:11.96:1.89:2.42:0.64. As well PPC purified fraction was found to be present in a molar ratio of Glu:Gal:Ara:Xyl:Rha:GalA:GluA = 35.16:22.10:24.95:10.44:3.69:3.10:0.56.

#### 2.4.4. FT-IR Spectroscopy

**Figure 7:**
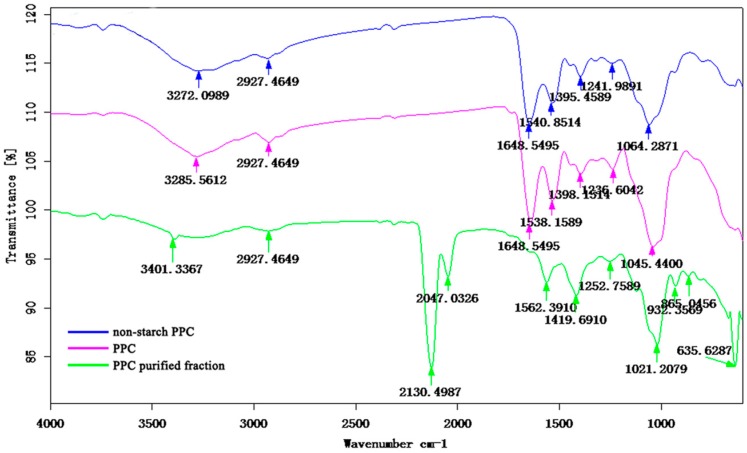
FT-IR of polysaccharides: PPC, non-starch PPC and PPC purified fraction.

The characteristic absorption of PPC, non-starch PPC and PPC purified fraction were identified FT-IR spectroscopy (Figure 7). The absorption peaks of PPC and non-starch PPC were roughly the same, and there were some differences with the PPC purified fraction. The infrared spectra of PPC (3285.56 cm^−1^); non-starch PPC (3272.10 cm^−1^) and PPC purified fraction (3401.34 cm^−1^) displayed a broadly-stretched intense peak at 3600–3200 cm^−1^ characteristic of hydroxyl and N-H groups. The small bands at around 2927.46 cm^−1^ (PPC, non-starch PPC, PPC purified fraction) were attributed to the C-H stretching and bending vibrations. The bands at 1648.55 cm^−1^ (PPC, non-starch PPC) were due to the bond stretching vibrations of C=O bonds in the acyl-amino group. The absorption peaks at 1538.16 cm^−1^ (PPC), 1540.85 cm^−1^ (non-starch PPC), 1562.39 cm^−1^ (PPC purified fraction) could be the bond asymmetric stretching vibrations of C=O bonds in the carboxyl group. The absorption peaks at 1236.60 cm^−1^ (PPC), 1241.99 cm^−1^ (non-starch PPC), 1252.76 cm^−1^ (PPC purified fraction) indicated the existence of O-H flexural vibrations. Each particular polysaccharide has a specific band in the 1200–1000 cm^−1^ region. This region is dominated by ring vibrations overlapped with stretching vibrations of (C-OH) side groups and the (C-O-C) glycosidic bond vibration [31].

Further, there were some special absorption peaks on PPC purified fraction. A characteristic peak at around 932.36 cm^−1^ (PPC purified fraction) indicates the β-configuration of the C-H deformation vibrations in the pyran ring [32]. The absorption at 865.05 cm^−1^ (PPC purified fraction) is typical of the dominant α- configuration in pyranose form [33]. Otherwise, the absorption peaks at 2130.50 cm^−1^ and 2047.03 cm^−1^ could be the impurity. All of above, the PPC has the typical polysaccharide absorptions.

## 3. Materials and Methods

### 3.1. Experimental Materials and Chemicals

Defatted peanut cake was purchased from the Lanshan Group (Liaocheng, Shandong Province, China). All chemicals used in this investigation were analytical grade and purchased from Beijing Chemicals Co., (Beijing, China).

### 3.2. Chemical Composition of Defatted Peanut Cake

The total sugar content was determined by a spectrophotometer (Model UV-1201, Beifen-Ruili Analytical Instrument (Group) Co., Ltd. Beijing, China) based on the reaction between sugars and phenol in the presence of sulfuric acid using glucose as a standard [34]. Nitrogen content was analyzed by a Kjeldahl Azotometer (Model KJELTEC2300, Foss Co., Copenhagen, Denmark) and the content of protein was calculated using a factor of 5.46. Crude fiber content was analyzed by a Fibertec system (Model Fibertec 2010, Foss Co.). Lipid content was analyzed by an automatic Soxhlet system (Model Soxtec 2050, Foss Co.). Ash and moisture were determined according to the methods described by AOAC 942.05 and AOAC 991.02.

### 3.3. Carbohydrate Composition of Defatted Peanut Cake

Fructose, glucose, sucrose, maltose, lactose were determined according to the methods described in AOAC 980.13. Stachyose was determined according to a described HPLC method. Total starch was determined according to the Megazyme method (Megazyme, 2011).

### 3.4. Hot-Water Extraction Procedure

Defatted peanut cake was ground in a high speed disintegrator to obtain a fine powder and dissolved in water to 8:1 (mL/g). In order to remove the protein from defatted peanut cake, neutral protease (activity of enzyme ≥ 6 × 104 U/g Beijing Solarbio Science & Technology Co., Ltd. Beijing, China) was added to solution at 45 °C and pH 7 for 150 min with constant stirring. After that, the mixed suspension was transferred to 90 °C water bath and left for 10 min to inactivate enzyme. Through centrifugation (4500× *g*, 20 min), the residual was collected [35].

Then the residue was added to deionized water with water-material ratios (mL/g) ranging from 10:1 to 30:1 while the temperature of the water bath ranged from 50 to 90 °C and was kept steady (within ±1 °C). The extraction time was changed from 1 to 5 h. After the entire extraction process, the mixture was centrifuged at 4500× *g* for 20 min and the supernatant was separated from insoluble residue.

Finally, ethanol with a final concentration of 75% (*v*/*v*) was added to the supernatant and the polysaccharides could be precipitated. After being left overnight at 4 °C, the precipitates were collected through centrifugation (4500× *g*, 20 min) and finally dried to get the crude polysaccharides. All tests were performed in triplicate. The extraction rate (%) of polysaccharides is calculated as follows:

Yield (%) = Dried Crude Extraction (g)/Defatted Peanut Cake (g) ×100%(2)

### 3.5. Experimental Design

Three levels using three variables; Box-Behnken Design (BBD) (Design Expert software, Trial Version 6.0.5, Stat-Ease Inc., Minneapolis, MN, USA) were applied to determine the best combination of variables for the highest extraction rate of polysaccharides from defatted peanut cake. Three variables considered for this research were extraction temperature (X_1_), extraction time (X_2_) and ratio of water to raw material (X_3_). The proper ranges of three variables were set based on single-factor experiments for the polysaccharides production [36].

Table 7 depicts three factors chosen for this study which were named as X_1_, X_2_, and X_3_ and the levels for each factor were coded with +1, 0 and −1 for high, intermediate and low value, respectively. The coded values of each factor were determined by the following equation:

I = 1, 2, 3(3)

where: X_i_ is the coded value; xi is the corresponding actual value; x0 is the actual value of the independent variable at the center point; and Δx is the step change of the variable. The complete quadratic equation used for the prediction is as follows:

Y = i = 1, 2, 3(4)

where: Y is the predicted response; X_i_ and X_j_ are the coded independent variables; β_0_ is the intercept coefficient; β_i_ is the linear coefficient; β_ii_ is the squared coefficient; and β_ij_ is the interaction coefficient.

Analysis of the experimental designed data and calculation of predicted responses were carried out using the SAS 9.0 software (SAS Institute Inc., Cary, NC, USA). The regression analysis of the data obtained was conducted to estimate the coefficient of the equation. Variance analysis (ANOVA) was used to determine the validity of the equations. The significance of each term in the equation represented the goodness of fit. The quality of the fit of the polynomial model equation was expressed by the coefficient of correlation (R^2^), and the significances of the regression coefficient were checked by F-test and *p*-value. Response surfaces were drawn to determine the individual and interactive effects of variables on the response. Additional confirmation experiments were subsequently conducted to verify the validity of the statistical experimental design.

### 3.6. Separation and Purification of Polysaccharides from Defatted Peanut Cake

Considering the possible effect of starch to the structure and purity, amylase was adopted. The dried crude PPC (5 g) was put in distilled water with α-amylase at a suitable pH and temperature (activity of enzyme ≥ 2 × 104 U/g, pH 5.5−7.0, 90 °C, Beijing Solar Bio Science & Technology Co., Ltd. Beijing, China) for 20 min. The mixed suspension was transferred to a 100 °C water bath and maintained for 10 min to inactivate the enzyme. The mixture was precipitated by the addition of ethanol in 75% (*v*/*v*) at room temperature and the precipitate was dried.

Then non-starch polysaccharide was purified by Sephadex G-100 filtration chromatography according to the reported method with little modifications. In brief, the non-starch polysaccharide solution (3 mL, 10 mg/mL) was applied to a column (2.6 cm × 70 cm) of Sephadex G-100 dextran. Then, the column was eluted with ultrapure water at a flow rate of 0.4 mL/min. The obtained elute was collected automatically (4 mL/tube) and the polysaccharides were detected by the phenol-sulfuric acid method. As a result, one PPC fraction was obtained. The fraction was collected, concentrated, dialyzed and dried for further research.

### 3.7. Characterization of Polysaccharides from Defatted Peanut Cake

#### 3.7.1. Determination of Total Sugar, Protein, and Starch Contents

The PPC, non-starch polysaccharide and PPC purified fraction were assayed for their total content of sugar, protein, and starch. Total sugar was determined by the phenol-sulfuric acid assay. Protein content was estimated from the binding of Coomassie Brilliant Blue G-250 to protein using a bovine serum albumin standard [37]. Total starch was determined according to the Megazyme method. Each sample was analyzed in duplicate.

#### 3.7.2. Molecular Weight Determination

The molecular weight of PPC purified fraction was characterized by size exclusion chromatography multi-angle laser light scattering (SEC-MALLS Dawn Heleos, Wyatt Technology Co., Santa Barbara, CA, USA) method. The sample (2.0 mg) was dissolved in distilled water (5 mL) and passed through a 0.45 μm filter. The conditions were mobile phase: 0.1 M NaNO_3_; flow rate: 0.5 mL/m in column temperature 45 °C [38].

#### 3.7.3. Analysis of Monosaccharide Composition

The non-starch PPC and PPC purified fraction (10 mg) were hydrolyzed with 4M trifluoroacetic acid (TFA) at 120 °C for 4 h in sealed glass tube. The acid was removed by evaporation with nitrogen. Monosaccharide composition was determined by high performance anion-exchange chromatography (HPAEC) combined with a pulsed amperometric detector with a gold electrode. A Dionex ICS-3000 system (Dionex, Sunnyvale, CA, USA) equipped with a CarboPacTMPA1 column (4 × 25 mm^2^) and CarboPacTMPA1 guard column (4 × 50 mm^2^) was used. As references, the following standard sugars were used: rhamnose (Rha), arabinose (Ara), galactose (Gal), glucose (Glu), xylose (Xyl), fructose (Fru), galacturonic acid (GalA) and glucuronic acid (GluA) [39].

#### 3.7.4. FT-IR Spectroscopy

The IR spectra of polysaccharides were determined using a TENSOR Fourier transform infrared spectro-photometer (Bruker, Madison, WI, USA). The sample was ground with spectroscopic grade potassium bromide (KBr) powder and then pressed into a 1 mm pellet for FT-IR measurement in the frequency range of 4000–400 cm^−1^ [31,40].

#### 3.7.5. Statistical Analyses

Statistical analysis was performed using SAS 9.0 software (SAS Institute, Inc., Cary, NC, USA). The results were presented as mean values ± standard deviation of at least three experiments. Paired *t*-test (levels of significance, 0.01 or 0.05) was used to evaluate the statistical significance of differences with *p* < 0.01 or 0.05 which were considered statistically significant.

## 4. Conclusions

Peanut yields are increasing year by year, especially in China. Large amounts of defatted peanut meal can be produced, and according to this research, the PPC can be comprehensively used, avoiding the waste of by-products.

Polysaccharides have many biological functions such as immunoreactivity, and anti-inflammatory activity, which can provide nutritional health protection for humans. The carbohydrate composition of defatted peanut cake was previously unclear before, so in this paper the content of heteropolysaccharides was calculated by subtracting the contents of monosaccharides, oligosaccharides, starch and fiber from the total sugars of defatted peanut cake. The hot-water technology was applied for the polysaccharide extraction from defatted peanut cake. Based on the single-factor experiments, response surface methodology (RSM) was used to estimate and optimize the extraction conditions: extraction temperature (°C), extraction time (h) and ratio of water to raw material (mL/g). The correlation coefficient (R^2^) for the model equation was 0.9995. Through the analysis of the second-order polynomial model, a maximum extraction rate of 34.49% was obtained under the following conditions: extraction temperature at 85 °C, extraction time for 3h, and ratio of water to raw material at 20:1 (mL/g). Under this condition, the mean experimental value of extraction rate was 34.97%, corresponding well with the predicted value (*p* > 0.05). The starch was removed from the PPC, which was further purified by Sephadex G-100 chromatography. The average molecular weight of the purified PPC fraction was 2.383 × 10^5^ Da. The polysaccharide was mainly composed of glucose (35.16%), galactose (22.10%), arabinose (24.95%) and xylose (10.44%). Characterization of the purified PPC fraction by IR analysis showed the typical absorptions of polysaccharides. Further studies on the precise chemical structures and biological activities of polysaccharide in the defatted peanut cake are needed.

## Figures and Tables

**Table 1:** Proximate composition of defatted peanut cake (wet weight) ^a^.

Main Components	Moisture	Ash	Protein ^b^	Carbohydrate	Lipid	Crude Fiber
Content (%)	4.54 ± 0.04	8.78 ± 0.01	47.28 ± 0.41	32.45 ± 0.30	3.40 ± 0.06	3.64 ± 0.01

^a^ All values are expressed on a grams per 100 g of defatted peanut cake, and data are reported as mean ± standard deviation of mean (*n* = 3). ^b^ N × 5.46.

**Table 2:** Box-Behnken design and the response values for polysaccharide extraction rate.

Run Order	Coded Levels			Extraction Rate (%)
	X_1_	X_2_	X_3_	
	Temperature (°C)	Time (h)	Water to Material Ratio (mL/g)	
1	−1	−1	0	2.89
2	−1	1	0	3.29
3	1	−1	0	20.11
4	1	1	0	23.45
5	0	−1	−1	4.60
6	0	−1	1	4.35
7	0	1	−1	6.23
8	0	1	1	4.54
9	−1	0	−1	5.53
10	1	0	−1	34.91
11	−1	0	1	15.72
12	1	0	1	22.85
13	0	0	0	14.23
14	0	0	0	13.99
15	0	0	0	14.13

**Table 3:** Analysis of variance for the response surface quadratic model for polysaccharides extraction rate.

Source	Degrees of Freedom	Sum of Squares	Mean Square	*F* Value	Pr > F
X_1_	1	682.44	682.47	5832.13	0.0001
X_2_	1	3.86	3.86	33.02	0.0022
X_3_	1	1.81	1.81	15.51	0.0109
X_1_X_1_	1	159.40	159.40	1362.17	0.0001
X_1_X_2_	1	2.16	2.16	18.47	0.0077
X_1_X_3_	1	123.77	123.77	1057.66	0.0001
X_2_X_2_	1	251.43	251.43	2148.68	0.0001
X_2_X_3_	1	0.52	0.52	4.43	0.0892
X_3_X_3_	1	3.23	3.23	27.56	0.0033
Model	9	1261.32	140.15	1197.65	0.0001
Error	5	0.58	0.12	12.75	0.0735
Total	14	1261.91			

**Table 4:** Fit Statistics for Y.

Source	Master Model	Predictive Model
Mean	12.72133	12.72133
R^2^	99.95%	99.95%
Adj. R^2^	99.87	99.87
RMSE	0.342079	0.342079
CV	2.689022	2.689022

**Table 5:** Optimum conditions and the predicted and experimental value of response at the optimum conditions.

Conditions	Extraction Temperature (°C)	Extraction Time (h)	Ratio of Water to Raw Material (mL/g)	Extraction Rate of Polysaccharides (%)
Optimum conditions	83.7	2.8	18:1	34.97 (predicted)
Modified conditions	85	3	20:1	34.49 ± 0.3 (actual)

**Table 6:** The contents of total sugar, protein and total starch ^a^.

Sample	Total Sugar (%)	Protein (%)	Total Starch (%)
PPC	87.67 ± 0.07	10.53 ± 0.22	60.92 ± 1.06
Non-starch PPC	44.40 ± 0.10	40.73 ± 0.10	4.19 ± 0.06
Purified fractions	82.46 ± 0.08	nd	nd

^a^ Each value is expressed as means ± standard deviation (*n* = 3); nd, not detected.

**Table 7:** The range of independent variables and their corresponding levels.

Independent Variables	Symbol	Coded Factor Level		
	Coded	−1	0	1
Temperature (°C)	X_1_	70	80	90
Extraction time (h)	X_2_	2	3	4
Ratio (mL/g)	X_3_	15:1	20:1	25:1

## References

[1] FAO FAOSTAT-Production-Crops. http://faostat.fao.org/site/567/DesktopDefault.aspx?PageID=567#ancor.

[2] Koji O., Satoshi T., Michiya K., Takanori M., Yasuhiro K., Junichi S. (2007). Efficacy of adjuvant immunochemotherapy with polysaccharide K for patients with curative resections of gastric cancer. Cancer Immunol. Immunother..

[3] Wu X., Mao G., Fan Q., Zhao T., Zhao J., Li F., Yang L. (2012). Isolation, purification, immunological and anti-tumor activities of polysaccharides from Gymnema sylvestre. Food Res. Int..

[4] Moro C., Palacios I. (2012). Anti-inflammatory activity of methanolic extracts from edible mushrooms in LPS activated RAW 264.7 macrophages. Food Chem..

[5] Chen G., Ma X., Liu S., Liao Y., Zhao G. (2012). Isolation, purification and antioxidant activities of polysaccharides from *Grifola frondosa*. Carbohydr. Polym..

[6] Chen Y., Luo H., Gao A., Zhu M. (2012). Extraction of Polysaccharides from Mango (*Mangifera indica* Linn.) Seed by response surface methodology and identification of their structural characteristics. Food Anal. Methods.

[7] Oosterveld A., Harmsen J.S., Voragen A.G.J., Schols H.A. (2003). Extraction and characterization of polysaccharides from green and roasted Coffea arabica beans. Carbohydr. Polym..

[8] Yin X., You Q., Jiang Z. (2011). Optimization of enzyme assisted extraction of polysaccharides from Tricholoma matsutake by response surface methodology. Carbohydr. Polym..

[9] Tian L., Zhao Y., Guo C., Yang X. (2011). A comparative study on the antioxidant activities of an acidic polysaccharide and various solvent extracts derived from herbal *Houttuynia cordata*. Carbohydr. Polym..

[10] Yan Y., Yu C., Chen J., Li X., Wang W., Li S. (2011). Ultrasonic-assisted extraction optimized by response surface methodology, chemical composition and antioxidant activity of polysaccharides from *Tremella mesenterica*. Carbohydr. Polym..

[11] Gan C.Y., Latiff A.A. (2011). Extraction of antioxidant pectic-polysaccharide from mangosteen (*Garcinia mangostana*) rind: Optimization using response surface methodology. Carbohydr. Polym..

[12] Sun Y., Liu J., Kennedy J.F. (2010). Application of response surface methodology for optimization of polysaccharides production parameters from the roots of *Codonopsis pilosula* by a central composite design. Carbohydr. Polym..

[13] Zhong K., Wang Q. (2009). Optimization of ultrasonic extraction of polysaccharides from dried longan pulp using response surface methodology. Carbohydr. Polym..

[14] Box G.E.P., Behnken D.W. (1960). Some new three level designs for the study of quantitative variables. Technometrics.

[15] Ferreira S.L.C., Bruns R.E., Ferreira H.S., Matos G.D., David J.M., Brand G.C. (2007). Box-Behnken design: An alternative for the optimization of analytical methods. Anal. Chim. Acta.

[16] Cobb W.Y., Swaisgood H.E. (1971). Roasted peanut flavor and its relation to growth environment. J. Food Sci..

[17] Mason M.E., Johnson B., Hamming M. (1966). Flavor components of roasted peanuts. Some low molecular weight pyrazines and pyrrole. J. Agric. Food Chem..

[18] Newell J.A., Mason M.E., Matlock R.S. (1967). Precursors of typical and atypical roasted peanut flavor. J. Agric. Food Chem..

[19] Basha S.M. (1992). Soluble sugar composition of peanut seed. J. Agric. Food Chem..

[20] Bryant R.J., Rao D.R., Ogutu S. (2004). α and β-galactosidase activities and oligosaccharide content in peanuts. Plant Foods Hum. Nutr..

[21] Zhu A., Tang L., Fu Q. (2015). Optimization of alkali extraction of polysaccharides from foxtail millet and its antioxidant activities *in vitro*. J. Food Biochem..

[22] Bo R., Ma X., Feng Y. (2015). Optimization on conditions of Lycium barbarum polysaccharides liposome by RSM and its effects on the peritoneal macrophages function. Carbohydr. Polym..

[23] Basedow A.M., Ebert K.H. (1977). Ultrasonic degradation of polymers in solution. Adv. Polym. Sci..

[24] Cui F., Xu H., Shu C., Xu Z., Tao W. (2006). Optimization of processing parameters for extraction of soluble *Grifola frondosa* polysaccharides by response surface methodology. Food Sci..

[25] Lorimer J.P., Mason T.J., Cuthbert T.C., Brookfield E.A. (1995). Effect of ultrasound on the degradation of aqueous native dextran. Ultrason. Sonochem..

[26] Shen A., Zhu Z., Zhang W. (2004). Studies on the extraction technology of water soluble polysaccharides from mulberry leaf. Canye Kexue.

[27] Sun F., Gu W., Ding X. (2006). Study on the extraction technology of polysaccharide from *Dioscorea opposite* Thunb. J. Food Sci. Biotechnol..

[28] Liang R.J. (2008). Optimization of extraction process of *Glycyrrhiza glabra* polysaccharides by response surface methodology. Carbohydr. Polym..

[29] Han J., Jiang X., Zhang L. (2011). Optimisation of extraction conditions for polysaccharides from the roots of *Isatis tinctoria* L. by response surface methodology and their *in vitro* free radicals scavenging activities and effects on IL-4 and IFN-γmRNA expression in chicken lymphocytes. Carbohydr. Polym..

[30] Yokota H., Mori K., Yamaguchi H., Kaniwa H., Saisho N. (1999). Monosaccharide composition analysis of pamiteplase by anion exchange chromatography with pulsed amperometric detection. J. Pharm. Biomed. Anal..

[31] Kacurakova M., Capek P., Sasinkova V., Wellner N., Ebringerova A. (2000). FT-IR study of plant cell wall model compounds: Pectic polysaccharides and hemicelluloses. Carbohydr. Polym..

[32] Coimbra M.A., Barros A., Barros M., Rutledge D.N., Delgadillo I. (1998). Multivariate analysis of uronic acid and neutral sugars in whole pectic samples by FT-IR spectroscopy. Carbohydr. Polym..

[33] Barker S.A., Bourne E.J., Stacey M. (1954). Infra-red spectra of carbohydrates. Part I. Some derivatives of d-glucopyranose. J. Chem. Soc. (Resumed).

[34] Dubois M., Gilles K.A., Hamilton J.K., Rebers P.A., Smith F. (1956). Colorimetric method for determination of sugars and related substances. Anal. Chem..

[35] Han B., Li Q. (2010). Extraction Optimization and Antioxidant Activity of Peanut Polysaccharides. Food Res. Dev..

[36] Ye Z., Wang W., Yuan Q. (2016). Box-Behnken design for extraction optimization, characterization and *in vitro* antioxidant activity of Cicer arietinum L. hull polysaccharides. Carbohydr. Polym..

[37] Bradford M.A. (1976). Rapid and sensitive method for the quantitation of microgram quantities of protein utilizing the principle of protein-dye binding. Anal. Biochem..

[38] Martínez H.B., Hortal A.R., Hurtado P. (2007). Laser desorption/ionization determination of molecular weight distributions of polyaromatic carbonaceous compounds and their aggregates. J. Mass Spectrom..

[39] Harazono A., Kobayashi T., Kawasaki N. (2011). A comparative study of monosaccharide composition analysis as a carbohydrate test for biopharmaceuticals. Biologicals.

[40] Peng H., Zhou M., Yu Z., Zhang J., Ruan R., Wan Y., Liu Y. (2013). Fractionation and characterization of hemicelluloses from young bamboo (*Phyllostachys pubescens Mazel*) leaves. Carbohydr. Polym..

